# Virtual Work Meetings During the COVID-19 Pandemic: The Good, Bad, and Ugly

**DOI:** 10.1177/10464964211015286

**Published:** 2022-06

**Authors:** Katherine A. Karl, Joy V. Peluchette, Navid Aghakhani

**Affiliations:** 1 The University of Tennessee at Chattanooga, Gary W Rollins College of Business, Chattanooga, TN, USA; 2 Lindenwood University, Saint Charles, MO, USA

**Keywords:** virtual meeting, videoconferencing, remote work

## Abstract

This study focuses on *the good, the bad and the ugly* of using videoconferencing for work-related meetings during the COVID-19 pandemic. Using a text mining process and qualitative content analysis of 549 comments posted to a LinkedIn online discussion board, we identified six key themes; three were tied to camera and microphone issues, two involved eating and meeting management issues, and one dealt with work-from-home issues. These themes are discussed in relationship to media naturalness theory and meeting science. Because widespread use of videoconferencing will likely continue, we provide guidance for workplace policies/practices and suggest directions for future research.

The COVID-19 pandemic and the resulting stay-at-home orders have led to significant changes in the way people work. One of these changes involves increased use of video conferencing as a means of communicating or holding work meetings. Zoom, for instance, had 10 million daily meeting participants in December 2019, but by April 2020, that number had risen to over 300 million (Evans, 2020). Other video conferencing platforms, such as Google Meet™ and Microsoft Teams, have also experienced significant increases in daily participants (Peters, 2020; Thorp-Lancaster, 2020). Furthermore, it is likely that the use of videoconferencing will continue long after the pandemic ends, as Gartner predicts that only 25% of business meetings will take place in-person by 2024 (Standaert et al., 2021).

Yet, for many, the increased use of videoconferencing has been challenging. For example, many users complain of *Zoom fatigue* or feeling mentally and physically exhausted by video conferencing (Fosslien & Duffy, 2020; Strassman, 2020). This exhaustion is due to several factors, one of which is prolonged direct eye gaze (Bailenson, 2020). In a normal face-to-face meeting, participants spend very little time looking directly into the eyes of one another, whereas in a video conference, individuals are typically staring more intensely at one another for the entire meeting (Strassman, 2020). Another factor is that the images of others on screen can appear too big and too close, triggering increased brain activity, biochemical changes, and physiological states that are associated with high alert and fight-or-flight (Morris, 2020). This is because the size and proximity of such images can violate our sense of personal space or cause us to subconsciously view them as threatening. Participants may also experience information overload as they attempt to focus on multiple faces at the same time, all in one-inch boxes that often jump from one position on the screen to another as different individuals speak (Morris, 2020). This is complicated by the fact that during virtual meetings, it is not just others’ faces that draw the attention of participants, but people or things visible in others’ backgrounds as well (Fosslien & Duffy, 2020). The chat function, although useful at times, can also add to participants’ information processing load, especially if the content detracts from the meeting (Wiederhold, 2020). Furthermore, seeing one’s own self-image can make users hyper-aware of themselves and their appearance, leading to the feeling of self-consciousness and always being *on* (Fosslien & Duffy, 2020).

Because videoconferencing is likely to become the preferred mode for business meetings and working from home may become permanent for many, a greater understanding of the potential challenges caused by videoconferencing is needed. To that end, the purpose of this study is to examine user opinions on the difficulties encountered with conducting business meetings from home via videoconferencing. In the following section, we review the relevant literature on videoconferencing and, then using media naturalness theory (Kock, 2004) and meeting science (Mroz et al., 2018), we provide a theoretical framework for understanding the difficulties inherent with videoconferencing as a communication medium.

## Literature Review

### Use of Videoconferencing for Workplace Meetings

While there is a growing body of literature on virtual teams (Dulebohn & Hoch, 2017; Gibbs et al., 2017) and the use of videoconferencing in education (e.g., Correia et al., 2020) and medicine (Fatehi et al., 2014; Weinstein et al., 2014), our focus is specifically on the use of videoconferencing for business meetings (e.g., Zoom, Microsoft Teams, Google Meet, GoToMeeting, Skype for Business). To date, research has examined the capabilities supported by videoconferencing and other meeting modes (e.g., face to face, audio conferencing), as well as the effectiveness of various meeting modes in achieving certain meeting objectives. For example, in comparison to face-to-face meetings, videoconferencing does not allow for life-size presence in a shared space, the transmission of haptic (touch) or olfactory (scent) cues (Standaert et al., 2016), the ability to observe what attendees are looking at, to see attendees’ body language and gestures, to have side conversations with one or more attendees, or to examine and/or manipulate specific physical objects (e.g., prototypes or samples) (Standaert et al., 2021). Similarly, research by Kuzminykh and Rintel (2020a) found that videoconferencing limited participants’ ability to understand the social dynamics of the group (i.e., who is important) and view communicative signaling, such as who was looking at whom.

Research has also examined participant engagement and multitasking behavior during videoconference meetings. For example, Kuzminykh and Rintel (2020b) found participants reported feeling lower motivation to engage both behaviorally and cognitively when participating in a meeting remotely versus face to face. Participants also noted that turning one’s video on or off was a crucial signal of engagement, with camera *on* signaling high engagement and camera off indicating low engagement. Cao et al. (2021) found multitasking to be a common behavior in videoconference meetings, with about 30% of meetings involving email multitasking. Many participants (32%) noted they were more likely to multitask when the video camera and microphone were turned off. Their findings also revealed that multi-tasking was more likely to occur during meetings that are large, long in duration, scheduled during the morning, regularly recurring, and perceived as less relevant. In terms of outcomes, while some participants (15%) mentioned that multi-tasking in meetings increased their productivity, a greater number (36%) mentioned negative outcomes, including losing track of meeting content (where the content was important), increased mental fatigue, and being perceived by others as rude, impolite, or disrespectful.

Two other recent studies focused specifically on the challenges and outcomes of the increased use of videoconferencing systems due to forced work-from-home mandates during COVID-19. Applying Gibson’s (1977) affordance theory perspective, Waizenegger et al. (2020) used interview data to identify the positive and negative effects of technology on team collaboration. Their results suggest that videoconferencing provided a social affordance or the opportunity to communicate with others and share ideas. However, while virtual meetings were generally welcomed by individuals living alone who craved social contact, working parents complained more of Zoom fatigue and having too many meetings or perceiving meetings as intrusive, with some noting increased role conflict due to the blurring of work-life boundaries.

Similarly, Hacker et al. (2020) used an affordance theory perspective to analyze Twitter tweets regarding the use of videoconferencing systems (e.g., Microsoft Teams, Skype, Zoom) during COVID-19. Using text mining, these researchers identified five major affordances and five constraints. For example, the use of videoconferencing allowed users the opportunity to communicate with social groups, engage in shared social activities with family and friends, attend events, pursue hobbies, and consume non-recreational services (e.g., webinars). The constraints included problems with the technology or incompetence in setting up the videoconferencing system, fear of being on camera, Zoom fatigue (being always *on)*, exposing one’s private living space, and lacking security (e.g., Zoom bombing).

### Meeting Science

When constructed well, meetings can provide a forum for creative thinking, discussion, debate, information sharing, problem solving and decision making. They can also help organizations meet important employee socio-emotional needs such as empowerment, engagement, affiliation, and perceptions of supervisor support (Christian et al., 2011; Yoerger et al., 2015). When poorly structured and managed, meetings can result in negative employee dispositions that lower employee perceptions of their work and well-being, as well as negatively impacting an organization’s bottom line (Rogelberg et al., 2014). For example, according to Doodle’s *State of Meetings Report* (2019), the cost of poorly organized meetings in 2019 was $399 billion in the United States.

Inspired by early work of Schwartzman (1986) who argued for the importance of studying meetings and talk in organizations, meeting science has evolved into a field of study and is defined as the systematic study of what happens prior to, during, and after meetings, in addition to how meetings fit into the context of organizations (Olien et al., 2015). Over the past twenty years, meetings research has focused on developing best practices for meeting design and composition, meeting structure and management of actions during meetings, and after-meeting considerations of participant satisfaction and decision outcomes (Mroz et al., 2018). These practices are an effort to address problems which frustrate meeting participants and to improve meeting effectiveness, such as calling a meeting only when necessary, including only those people whose expertise and knowledge is required, preparing and following an agenda, starting the meeting on time, avoiding distractions and multitasking, and actively encouraging everyone to participate (Lehmann-Willenbrock & Allen, 2018; Mroz et al., 2018). Research that integrates the meeting science and virtual teamwork literatures identifies similar practices for effective virtual meetings (Allison et al., 2015) including: selecting a meeting facilitator, choosing the appropriate mode of communication (e.g., phone, email, videoconference), setting meeting norms, establishing team roles, recognizing time zone and cultural differences, and following up with action items after the meeting.

### Media Naturalness Theory

Using Darwinian evolutionary principles, media naturalness theory (MNT) assumes that the human brain has evolved over time to facilitate face-to-face communication and that the more similar a communication mode is to face-to-face communication, the more natural it is and the lower the cognitive effort required to use it (Kock, 2004). MNT identifies five key characteristics of media naturalness: (1) co-location, or the participants are located in a common physical space, (2) synchronicity which allows for immediate and spontaneous exchanges of communicative stimuli, (3) the ability to observe and convey facial expressions, (4) the ability to observe and convey body language, and (5) the ability to convey and listen to speech. As noted earlier, previous research (Standaert et al., 2016, 2021) suggests that videoconferencing lacks some of these characteristics (e.g., co-location, body language, and possibly facial expression if the video is turned off or there are several participants on one screen). Consequently, communication between individuals using videoconferencing is less natural and more cognitively demanding.

While MNT has much in common with other theories such as media richness theory (Daft & Lengel, 1984) and social presence theory (Short et al., 1976), these earlier theories have not always been successful in explaining choice, satisfaction, or effectiveness of computer-mediated modes of communication (e.g., Dennis & Kinney, 1998; Trevino et al., 1990). One assumption made by MNT that is particularly relevant to videoconferencing is regarding the increased cognitive effort required to use non-face-to-face modes of communication. Media Richness Theory and Social Presence Theory assume that more is better. In other words, the richer the communication medium or the more social presence that exists, the better the communication. In contrast, by focusing on the human biological communication apparatus (which includes the elements of the brain and body that are used in communication interactions such as the vocal tract, facial muscles and visual and auditory organs; Kock, 2004), MNT assumes that a communication medium can be too rich and lead to information overload, causing individuals to be overwhelmed, dissatisfied, and less productive (Hantula et al., 2011).

### Research Questions

Because of the widespread use of videoconferencing as the primary medium for work communication during the COVID-19 pandemic and the likelihood that it will continue to be widely used in the workplace, it is important to understand what about videoconferencing is working, and what is not working, for individuals who are working remotely. In other words, the focus of this study is on the “good, the bad, and the ugly” of using videoconferencing for work-related meetings, where “bad” refers to anything that deters from the meeting process and “ugly” is something that is visually distracting to participants and impedes meeting effectiveness. More specifically, based on our literature review, we are pursuing the following research questions:

**RQ1:** In what ways does the use of videoconferencing enhance the user experience or meeting effectiveness?
**RQ2:** In what ways are the frustrations faced by virtual meeting participants similar or different from those experienced by participants in face-to-face meetings?
**RQ3:** Are these frustrations related to the technology, participant behavior, or the situation (e.g., COVID-19)?

## Method

### Research Design and Sample

The data for this study was collected from a LinkedIn website dealing with Zoom or video meeting etiquette, entitled “Chewing while on Zoom? Oh dear.” In addition to the title article, LinkedIn members posted four additional articles on the site including: “When It Comes to Zoom Etiquette, What’s Your Pet Hate?,” “Seven Rules of Zoom Meeting Etiquette from the Pros,” “Video Meeting Etiquette,” and “Zoom Call Etiquette.” All comments posted on the website (*N* = 549) were cut and pasted into an Excel spreadsheet and included in the analysis. All comments were posted in July/August of 2020.

### Analyses

To extract the topics from the comments collected from LinkedIn, we used Latent Semantic Analysis (LSA) in SAS Enterprise Miner which is a powerful text mining method capable of uncovering the underlying semantic concepts (i.e., topics) in a corpus. LSA is an algorithm based on singular value decomposition (SVD) to reduce the dimensional matrix of latent semantics to extract knowledge from the textual corpus (Evangelopoulos et al., 2012; Shen & Ho, 2020) and has been widely used in various contexts such as analysis of textual data in computer-mediated communication (Cao et al., 2011; Xu, 2020) and quantitative literature reviews (Jeyaraj & Zadeh, 2020; Kulkarni et al., 2014). Given its effectiveness in analyzing unstructured textual data, we used the LSA topic modeling method to uncover underlying topics related to participant frustrations with Zoom videoconferencing.

First, we preprocessed and cleansed the comments by eliminating numbers and punctuations from the data. Because some words (i.e., stop words such as “a,” “all,” or “the”) are generally not useful in identifying the discussed topics, we used the Standard English stop word dictionary to exclude these from the data and reduce its dimensionality. Consistent with Jeyaraj and Zadeh (2020), various actions such as tokenization, lemmatization, stemming, spell-checking, and synonyms were performed. We also ignored the different parts of each to further reduce the dimensionality of the data.

Following Shen and Ho (2020), a term-by-frequency matrix was created to parse the comments to a collection of terms. In a term-by-frequency matrix, each column of the matrix represents a unique word that appears across all comments, and each row refers to each comment. Each cell in the term-by-frequency matrix represents the number of times that a term (column) appears in a particular row (comment). Using term-by-frequency matrix weighting alone cannot effectively distinguish different patterns of the comments (Cao et al., 2011) because a term that appears commonly in a comment may appear in other comments as well. For instance, in LinkedIn, the term “zoom” appeared in many comments, covering different topics or challenges related to Zoom video conferencing. To address this problem, the term frequencies were adjusted by the TF-IDF weighting scheme (term frequency-inverse document frequency).

Because the matrix created by the TF-IDF weighting scheme is very large and sparse, we eliminated the terms that appeared in less than four comments. We also applied the Singular Value Decomposition (SVD) method to reduce the dimensionality of the data while still retaining most of the meaningful information (Jeyaraj & Zadeh, 2020). The final step involved setting the underlying dimensions (topics) in the LSA algorithm. Following the recommendations of Evangelopoulos et al. (2012), we used qualitative assessments to link the results to underlying theories. In doing so, the authors performed a qualitative content analysis and identified 11 possible topics in the corpus, using this number as the baseline to run the LSA algorithm. After a couple of iterations and qualitative analysis of classified comments, the authors came to an agreement that the best degree of separation was when the LSA algorithm was run with six predefined topics.

## Results

After reviewing all comments appearing under each of the six topics, we identified labels for each. These six topic labels included camera on versus off, lurking (*N* = 130), meeting management issues (*N* = 113), camera issues (*N* = 111), eating during meetings (*N* = 99), microphone issues (*N* = 93), and work from home issues (*N* = 93). Below, we review each of these themes, and tie the findings to media naturalness theory and the meeting science literature.

### Camera On Versus Off, Lurking

Many contributors felt strongly that videoconference participants should leave their camera on during meetings and, that not doing so, impeded communication or was a sign of disrespect. For instance, one contributor stated,

It’s a bit like going to a meeting and sitting there with a bag over your head. It makes it hard for everyone to engage in a meaningful way. 55% of our communication is visual. There are circumstances that may require your video to be off but if you can have it on, it makes for better communication.

Likewise, another stated, “it’s always nice to see the person on the screen since it helps in improving the quality of the interactions. Non-verbal cues are just as critical as the spoken word.” These comments are consistent with Media Naturalness Theory in that the contributors viewed facial expressions as key to effective communication. Others emphasized that having the camera on was a means by which coworkers could enhance their relationships with their coworkers. For example, one stated,

I have learned so much about my coworkers through this. I’ve virtually met so many of their children, spouses, partners, etc. So, in a way, it’s been refreshing. We have shaken off the hierarchy of the workplace and it has been more human. I love it.

Still others voiced the opposite opinion believing the camera should be off for a variety of reasons including Zoom fatigue, lack of bandwidth, discomfort with being on camera, and to prevent their home life (visible in their background) from distracting other participants. As an example, one contributor stated, “not everyone has the bandwidth to use video consistently.” Another stated, “Not being on video does not make you a “lurker.” Some people love being on video, some of us do not.” These comments are also consistent with media naturalness theory. Lack of bandwidth interferes with the meeting transmission, thereby disrupting the synchronicity of communication exchanges and often results in participants turning off their camera and using audio only. Discomfort with being on-camera is another issue. In face-to-face communication, individuals see those to whom they are speaking, they do not see themselves. Many find that seeing their own image on their computer screen is unnatural and distracting. There was also recognition among some contributors that the pandemic may be impacting individuals’ decisions to have their camera off, such as to prevent others in their environment (e.g., children, spouse) from disrupting the meeting or distracting other meeting participants. For example, one stated, “To anyone who chooses “not turning video on,” I encourage you to “assume positive intent” . . . they may have kids running around.”

### Meeting Management Issues

This theme included comments related to characteristics of “bad” meetings and problems with meeting management. Comments related to “bad” meetings included concerns about participants who show up late, lack of an agenda, meetings are too long, too many meetings, back-to-back meetings, and participants who multi-task. An example of a contributor who complained of too many meetings or back-to-back meetings is as follows, “Multiple zoom meetings. . . I didn’t like multiple in person meetings, now I can spend an entire day on virtual calls. There is no lunch time or break time, just jump from meeting to meeting.” Existing research in the Meeting Science literature shows that meeting load, or the frequency and time spent in meetings (especially bad meetings), can increase employee stress, fatigue, and perceived workload (Luong & Rogelberg, 2005; Rogelberg et al., 2006).

Multitasking during virtual meetings is also viewed negatively, as indicated by the following comment, *“*those who multi-task during a zoom meeting are not giving their utmost attention to the meeting. . . . Let the organizers put forward ground rules on the invitation of attendance.*”* Previous research on face-to-face meetings suggests that both meeting satisfaction and effectiveness are enhanced when participants avoid engaging in activities unrelated to the topic of the meeting (Odermatt et al., 2018). However, there are those who see multitasking as necessary, given the volume of virtual meetings that they are now dealing with. This is reflected in the following comment, “what about the fact that I’m averaging 3 to 4 meetings PER DAY when previously I would maybe have 3 to 4 in A WEEK? If I am not multitasking, I am losing productivity.”

A contributor comment that addressed meeting management issues, with suggestions for improvement, is as follows:

We should also learn how to make the most out of Zoom meetings. What I am learning: (1) Always set an agenda and goals for the meeting. Are you merely disseminating information so you wouldn’t need people to be turning on their videos or participating unless they need to ask questions? Or is it a meeting wherein inputs of attendees will have bearing on decisions to be made? (2) Set the time and manage the time well. Zoom fatigue sets in if meetings go beyond 1.5 hours and one has to be in those meetings four times a day. (3) Consider the number of attendees. Just because zoom premium account allows 200 participants in a meeting room, it doesn’t mean you maximize the room space. Of course webinars are a different matter. (4) Online meetings is still part of work, it requires professionalism. Being on time and coming to meetings prepared are still required.

To support meeting management, some contributors noted that the chat function and polling could be used to enhance participant involvement and engagement in the meeting. As an example, one contributor stated, “There’s also an onus on people running zoom meetings to keep the audience engaged, e.g., by using polls and questions in the chat box.” Taken together, these comments reflect the importance of attention to effective meeting management, ranging from how virtual meetings are planned and scheduled to how they are conducted (Lehmann-Willenbrock et al., 2018).

### Camera Issues

Several contributors voiced frustration about camera issues including the camera angle, proximity of the camera to a participant’s face, bad lighting, participants who do not look at the camera, and participants who walk around with the camera. For example, one participant stated,

my pet peeve is floating heads at the bottom of the screen. Tilt your camera so your head is near the top of the screen and you basically fill the frame. . . .Second is bad lighting. We’re all doing this regularly now, so read some articles on how to get decent lighting for your video calls. Good video setup is the new business suit.” Another person said, “I feel like I’m on a roller coaster when people walk with the camera on, it’s so distracting, and you can’t help but be drawn to watching it!

These complaints are consistent with media naturalness theory, which assumes any form of communication that moves away from face-to-face interaction leads to extra cognitive effort. Face-to-face communication involves being able to make direct eye contact and to see the other persons’ entire face.

### Eating During Meetings

Most of the comments in this theme focused on meetings that were scheduled over the lunch hour and, while many were irritated by participants who were eating on camera, others were more accepting and believed that this was necessary. To illustrate, one contributor stated,

I had a manager that would book google meetings and then be eating the whole time I felt like I was not valued and that the meeting was a waste of her time. . . .Not very professional! Eat before you have a call!

Others voiced the opposite opinion. For example,

If you schedule a meeting over lunch, you should be ok with people eating during that call. People need to eat.” Likewise, another stated: So, please STOP scheduling meetings during lunch hour! . . .Previously at the workplace, if the occasional meeting needed to be scheduled at noon, lunch was provided for participants. Don’t I deserve a break to eat? If you want me on a call during the lunch hour, prepare to see me eat!

Still others recommended that individuals turn off their camera if they must eat. A sample comment is:

If people have to eat during a video call, turning off the camera until they are done can save the awkwardness of having to watch somebody eat. Back when we all sat in conference rooms together, lunch meetings were common and nobody thought it was so awful to watch their colleagues chew–mostly because they didn’t watch.

Those who complained about their coworkers eating during meetings clearly saw the behavior as distracting and rude. In contrast to many face-to-face meetings, it is likely that virtual meetings scheduled over the lunch hour had no expectation or norm that everyone would be eating. Also, due to the positioning of cameras, others’ faces appear much closer and less natural in virtual meetings, so that if someone is eating, it is highly visible. Given the findings of Odermatt et al. (2018) on uncivil meeting behavior, we suggest that such individuals would likely experience lower meeting satisfaction and perceive reduced meeting effectiveness.

### Microphone Issues

Another frustration faced by participants in virtual meetings was coworkers who failed to mute their microphones. Sample comments include: “Not muting. Echoes and background noises can completely drown out the speaker, so keep it muted until you’re ready to speak” and “I really dislike when people have a commotion on their end and don’t mute themselves. I don’t want to hear their dogs, kids, or arguments.

These comments are also supported by media naturalness theory since the ability to convey and listen to speech is a key characteristic of face-to-face communication. Furthermore, Kock (2004) argues “that the ability of a medium to support the use of speech is likely to be significantly more important than all of the other naturalness elements” (p. 334). When individuals fail to mute their microphones, other participants hear only the background noises and not the speaker, losing their ability to listen to speech.

### Work-from-Home Issues

This theme was largely comprised of comments related to the unrealistic expectations (i.e., a professional workspace free from distractions) that employers had regarding working from home during a pandemic. As an example, one contributor stated,

We should be mindful that WFH is not like it used to be. In households where there is more than one person . . . there is no way to control some background distractions or noise. You now have all working from home with kids learning from home . . . there is now a new normal as it pertains to working from home and flexibility, empathy, understanding for others’ situations is key.

This comment clearly reflects the contributor’s frustrations that workplace meetings (traditionally face-to-face) and videoconference meetings, especially during a pandemic, are not the same. Likewise, another contributor wrote,

A global pandemic creates a scenario in which mass amounts of people who’ve never or rarely worked from home now have to adjust to doing so, often in spaces not bought or rented for doing so, and often with a partner in the same situation.

While most comments in this theme agreed that meeting etiquette is important and necessary in the workplace, the comments cited above, as well as many others, imply that the COVID-19 pandemic has created a unique situation which, at least in the short run, may require some leniency and understanding about others and their ability to control distractions in their work/home environment.

## Discussion

To answer the first research question we posed earlier, the results of our analysis suggest that some individuals believe that videoconferencing is, in some ways, better than face to face meetings. The perceived positive benefits include the use of polling, the chat function, and the ability to enhance relationships by seeing, and thereby learning, more about coworkers’ personal lives (i.e., their home environment). In response to our second research question, we found that many of the frustrations faced by participants in videoconference meetings are consistent with those experienced by participants in face-to-face meetings. Starting late, lack of an agenda, having meetings that are too long, and having participants show up late or multi-task were mentioned in comments posted by contributors. These are all problems that the meeting science literature has identified in face-to-face meetings and have been found to reduce both meeting satisfaction and effectiveness. However, our results showed there were also important differences in the experiences faced by videoconference participants working from home.

In response to our third research question, these frustrations were associated with all three of the expected factors: technology, participant behavior, and the unique situation resulting from the COVID-19 pandemic. An examination of our text mining results suggests that most contributors’ frustrations stem largely from issues related to how others were using the technology. Over 60% of the total comments focused on camera issues (e.g., angle), whether the camera was on or off, and microphone issues (e.g., failing to use mute). Another common comment was displeasure with having to watch others eating on camera. However, several stated that eating was often necessary due to many meetings being scheduled over the lunch hour. Some contributors also noted that seeing or hearing others’ background noises was distracting. This was largely a result of the COVID-19 pandemic in which employees found themselves forced to work from home and, at the same time, the home had become the school, playground, sports facility, and sanctuary.

### Practical Implications

With the initial onset of the COVID-19 pandemic, many employees had to learn almost overnight how to use videoconferencing, and our findings suggest many appear to have merely “muddled through.” Given research evidence showing that employee satisfaction with meetings is a distinct component of their overall satisfaction and a significant source of job-related stress and well-being (Lehmann-Willenbrock et al., 2018; Rogelberg et al., 2006), it is important for organizations to take the time to properly train their employees on how to use videoconferencing, including the standard protocols about the use of the microphone (e.g., mic off when not speaking) and proper camera/screen positioning. Such training should not only include the key features of the platforms used for virtual meetings (e.g., Zoom, Microsoft Teams, Google Meet), such as screen sharing and break out rooms) but new features of videoconferencing platforms or apps that enhance meeting effectiveness. For example, Krisp is a third-party app that cancels out background noise, and Muzzle silences pop-up notifications during screensharing (Kelly, 2020).

Our findings also suggest that many employees were unaware of social norms or meeting etiquette, thus organizations should provide expectations about how employees should present and/or conduct themselves in virtual work meetings. In addition, employees should be provided with training on ways to improve meeting structure by utilizing an agenda and establishing ground rules on meeting facilitation (e.g., use of the chat feature, raising one’s hand to speak). Managers and senior organizational leaders play a key role in reinforcing the importance of this training, as well as modeling appropriate meeting structure and behaviors. Because some of the comments in this study cited problems with Zoom fatigue and back-to-back meetings, organizations should carefully consider whether videoconferencing is being overused. In some instances, an email, phone call, or use of a messaging system (e.g., Slack) may be more effective.

### Limitations and Future Research

While our research has contributed to the literature by highlighting the frustrations participants have with using videoconferencing platforms for work-related meetings, there are some notable limitations to our study. First, more participant information would have been helpful in determining whether frustrations differ for participants based on their occupation, managerial status, marital and family status, type of meeting (e.g., organization-wide versus team), or hours spent per day in videoconference meetings. Secondly, those who contributed the comments in our analysis were not randomly selected, rather they were self-selected in that they made the choice to leave comments on the LinkedIn site. It is possible that their opinions may not be representative of all videoconference participants. However, our intent was not to test existing theory, but instead to tie our findings to relevant theories and provide direction for future research. In part, our findings contribute to the meeting science literature by identifying issues that are unique to videoconferencing. Using a lens of media naturalness, this study also provides new insight into many of the specific challenges with using this mode of communication (e.g., Zoom fatigue, lurking, background distractions). Yet, much remains unknown regarding how to maximize the satisfaction, engagement, and effectiveness of videoconferencing as means for work communication. Given that widespread use of this communication medium is expected to continue in the future, we believe that it is important to address that gap by providing an agenda for future research. More specifically, we developed three avenues (the good, the bad, and the ugly) to guide future research (see Table 1).

**Table 1.** Future Research Agenda on Videoconferencing and Potential Research questions.

Topic	Potential research questions
The good	
Chat function	Are introverts more likely to participate using the chat function?
	Are meetings more efficient when participants use the chat function as opposed to speaking?
Polling	To what extent does the use of polling improve meeting process and outcome satisfaction?
Relationship building	To what extent does seeing coworkers’ home and/or family life improve relationships among coworkers? How might this be used to build culture and engagement?
Visibility	Does increased use of videoconferencing compensate for lack of visibility when it comes to a remote worker’s mentoring opportunities and career progression?
Organizational culture, trust, cohesion	How can videoconferencing best be used to instill corporate values, culture, trust and group cohesion?
The bad	
Failing to mute	What is the best way to eliminate issues of poor microphone management during virtual meetings? Should the facilitator have sole control of who speaks?
Ineffective and/or inappropriate use of video camera	To what extent does camera angle, lighting, and not looking at the camera influence others’ impressions?
	What is the best way to eliminate these behaviors? Is training needed?
Poor planning and scheduling	How can scheduling virtual meetings be better managed to reduce the problem of back-to-back meetings with little or no opportunity for breaks?
	To what extent are unscheduled or impromptu virtual meetings seen as a challenge/interruption when compared to how these are viewed in the face-to-face work environment?
	To what extent is entering a virtual meeting early, and engaging in pre-meeting talk, important to meeting satisfaction and effectiveness?
	Is videoconferencing being overused? Under what circumstances is videoconferencing most appropriate?
Lurking	To what extent do lurkers impact the meeting satisfaction and engagement of others? Is lurking contagious?
	Are those who are highly self-conscious more distracted by their self-image and therefore less effective in video conferencing if their camera is on? Do they experience more stress?
	To what extent are those with their camera off not asked for their input or invited to participate?
	What would be employees’ reactions (perceptions of fairness/justice) to being told they must have their camera on?
	To what extent does having one’s camera off affect others’ perceptions of that individuals?
	Is personality or gender related to lurking?
Multi-tasking	What is the impact of multi-tasking on others’ perceptions, satisfaction, and engagement during virtual meetings? Is multi-tasking contagious?
	To what extent do meeting participants believe that others are multi-tasking when their cameras are turned off?
Gender inequity and exclusion	Are women interrupted or drowned out in conversation as much or more in virtual meetings as they are in face-to-face meetings?
	Do women experience lower meeting satisfaction and meeting effectiveness as compared to men?
Dominance	Compared to face to face meetings, are high status individuals less likely to dominate the discussion?
The ugly	
Eating during meetings	Should all on-camera eating be banned from meetings?
	Does observing others eating during a virtual meeting lead to process loss? If everyone is eating on-camera, is it less irritating or distracting to meeting participants?
Participants’ background	How does seeing participants’ backgrounds (messy vs. tidy, real vs. fake or blurred, bedroom, kitchen vs. office) impact others’ meeting satisfaction and effectiveness?
	To what extent does having children or others appear in the background during virtual meetings impact the meeting satisfaction of others?

While most of our proposed research questions relate to our findings, others expand on existing research. For example, a concern of remote workers is that lack of visibility will lead to slower career progression and fewer mentoring opportunities (Raghuram et al., 2019). We recommend that future research examine whether increased use of videoconferencing may compensate for lack of visibility. Existing research also shows that when high-status or highly influential group members dominate a group discussion, it decreases participation by other members and lowers meeting satisfaction (Mejias, 2007). In videoconferencing, status differences are less evident (i.e., everyone appears in a one-inch box in a random location on the screen) and evaluation apprehension may be lessened due to reduced nonverbal cues. Thus, it is recommended that future research examine whether dominance by influential members may occur less in videoconference meetings when compared to face-to-face meetings. Gender differences should also be examined, as some women find themselves frequently interrupted and spoken over during meetings (Gupta, 2020).

In a face-to-face work setting, teams often have impromptu meetings as they pass each other in the hall or at the water cooler (Stray & Moe, 2020). Since some videoconferencing platforms (e.g., Microsoft Teams) allow team members to instantly connect via video, we recommend future research examine the extent to which these unscheduled videoconferences are perceived as an interruption, leading to increased feelings of fatigue and workload. Finally, many organizations have started to look beyond the pandemic and are being proactive and intentional about determining what their remote work strategy will be going forward. Of concern to many is how they can continue to use videoconferencing as a means of instilling their corporate culture and building trust and cohesion within work groups (Alexander et al., 2020).
